# “I don’t want to be a victim again”: the impact of repeat assault on police officers

**DOI:** 10.3389/fpsyg.2023.1145944

**Published:** 2023-05-18

**Authors:** Louise Davidson, Amelia Dennis, Andriana Theodoropoulou, Holly Carter, Richard Amlôt, Ian Hesketh

**Affiliations:** ^1^Behavioural Science and Insights Unit, UK Health Security Agency (UKHSA), London, United Kingdom; ^2^National Forum for Health and Wellbeing at Work, The University of Manchester, Manchester, United Kingdom; ^3^Workforce Development, College of Policing, Ryton on Dunsmore, United Kingdom

**Keywords:** police officers, mental wellbeing, repeat assaults, occupational support, leadership, physical wellbeing

## Abstract

**Introduction:**

The frequency of assaults on police officers in the United Kingdom is rising and evidence suggests that exposure to work-place violence can negatively impact wellbeing, for example, increased perceived stress, feeling worn out and tired, and emotional exhaustion. Despite the prevalence of assaults on police officers, little research has examined the impact of repeat assaults on officers’ wellbeing.

**Method:**

For the current study, 12 semi-structured interviews were conducted to investigate the impact of repeat assaults on wellbeing and occupational outcomes in police officers and staff, including impacts on their mental and physical heath, impacts on their work, the impact of prior assaults on future assaults, and what support they were provided with.

**Results:**

Findings indicate that repeat assaults had a negative impact on participants mental and physical wellbeing. Furthermore, a lack of support both from management and peers within the police force was found to further exacerbate the impact of repeat assaults. However, the provision of support was also identified as a mitigating factor when it was available and provided to participants which helped to protect participants from some of the negative impact of repeat assaults.

**Discussion:**

Findings provide a unique in-depth perspective into police officers’ experiences following repeat assaults, which can in turn inform national policies and help tailor effective support services within the police force.

## Introduction

Police officers in the United Kingdom regularly face violence from the public and the number of assaults on police officers in the United Kingdom each year is increasing (Office for National Statistics, 2022). Accordingly, in the United Kingdom, in the year ending March 2022, there were over 41,000 assaults on police officers, with 11,730 resulting in injury (Office for National Statistics, 2022).

Whilst a systematic review of the relationship between organizational stressors and mental wellbeing within police officers identified 36 different organizational stressors, including organizational culture, job pressure, and long working hours that were related to mental wellbeing outcomes among police officers (Purba and Demou, 2019), the impact of assaults on mental wellbeing outcomes receives little attention in the literature. Because of the large number of assaults that police officers experience in their day-to-day job (Office for National Statistics, 2022), it is important to understand the impact of assaults (i.e., the specific *ways* assaults have an effect, either positively or negatively) on their wellbeing and work, as well as the support that they are offered. This is to ensure police officers are adequately supported. Evidence suggests that exposure to work-place violence can negatively impact wellbeing (Mueller and Tschan, 2011). Indeed, previous research has shown that violence and injuries sustained in the line of duty can lead to increased perceived stress, feeling worn out and tired, and emotional exhaustion (Santos et al., 2009; West et al., 2017; Wolter et al., 2019). In addition, research has also shown that assaults in the line of duty are related to increased psychological distress due to a fear of being assaulted again in the future (Leino, 2013). As well as negatively impacting wellbeing, workplace assaults can reduce job investment (Van Emmerik et al., 2007) and increase job stress, in turn reducing job satisfaction and commitment (Manzoni and Eisner, 2006). Furthermore, exacerbating this negative impact of assaults, legal proceedings surrounding assaults have been shown to also put police officers at increased risk for posttraumatic stress disorder (Ellrich and Baier, 2017) due to legal proceedings potentially being seen as secondary victimization (e.g., Orth, 2002), or fear of job loss in the case of the police officer being convicted of any wrongdoing (Ellrich and Baier, 2017).

Yet, despite evidence demonstrating the negative impacts of repeat assaults on officers’ wellbeing, in contradiction to this one study found that physical violence did not predict posttraumatic stress disorder (Brewin et al., 2022). However, upon further exploration recent research suggests this could be explained by the level of support provided to the officers following their assault, with peer support having been identified as a coping mechanism for posttraumatic stress disorder among police officers (Foley et al., 2022). In addition to peer support, organizational support has been shown to predict police officer willingness to use support services following stress (Tucker, 2015). The impact of assaults may also be reduced by the provision of preparatory sessions, where police officers are specifically trained and prepared for violent attacks as these sessions have been shown to increase perceptions of control and competence (Ellrich and Baier, 2017). Based on this evidence, there are ways in which the negative impact of assaults may be mitigated, and thus it is crucial to understand more about any potential mitigating factors, how they can be applied, and what effect they have on officers’ wellbeing following repeat assaults. As such, below we introduce an initiative called Operation Hampshire, which aims to provide strategy, processes, and guidance to better respond to assaults on police officers and staff.

### Operation Hampshire

Operation Hampshire is a National Police Wellbeing Service (NPWS) initiative which provides a comprehensive framework in the response to assaults on police officers, incorporating five key themes: (i) supervision, (ii) wellbeing, (iii) communication, (iv) investigation, and (v) criminal justice. A key area of focus identified by Operation Hampshire is the impact of repeat assaults. Given the potential adverse impacts of experiencing an assault, it is important to identify the impact of repeat assaults on police officers. To the best of our knowledge, little research has been conducted into the impact of repeat assaults on police officers’ wellbeing, though one study demonstrated that the frequency of work-related violence among police officers is associated with greater psychological distress and fear of future assaults (Leino, 2013). Based on this, albeit limited evidence, police officers’ experiencing repeat assaults is therefore likely to worsen the impact of assaults on their wellbeing. Repeat assaults may also affect factors such as work confidence and risk appetite, as previous research has found a positive association between experiencing assaults and use of force (Manzoni and Eisner, 2006). Furthermore, it is not only important to understand the impact of repeat assaults, but it is also important to understand the various factors that may mitigate or exacerbate the impact of repeat assaults in order to provide sufficient support to police officers’ to minimize any potential negative impacts on their wellbeing.

### The current study and aims

The current study investigated the impact of repeat assaults on wellbeing and occupational outcomes, using qualitative interviews with police officers who had experienced repeat assaults by members of the public in their line of work within the last year. More specifically the four key aims of the study were to:

1. Identify the impact of repeat assaults on police officers’ mental wellbeing and physical health.
2. Identify the impact of repeat assaults on aspects of police officers’ occupation (such as job satisfaction and performance).
3. Explore how prior experiences of assault might affect subsequent experiences.
4. Explore factors which may mitigate or exacerbate the impact of repeat assaults.

## Method

### Participants

Twelve participants took part in a semi-structured interview. All participants were employed in the Police Service at the time of interview—11 participants were police officers, and one participant was a Police Community Support Officer. Most participants were male (male, *n* = 10; female, *n* = 2) and ages ranged between 20 and 43 years old (*M* = 29.08; SD = 6.20); see Table 1 for participant demographics. Participants completed a screening questionnaire which included basic demographic questions (e.g., job role, age, gender, ethnicity) and questions about workplace assaults in the last year (e.g., number, injuries, and type of assault). Participants were invited to take part in an interview if they were over 18 years old and if they had experienced two or more assaults in the last year. However, despite reporting two assaults in the last year during the screening survey, one participant stated during the interview that they had only experienced one assault; this participant was not removed from analysis.

**Table 1:** Participant demographics.

	Frequency	Percentage
Age	*M =* 29.08	SD *=* 6.20
Gender		
Female	2	16.67%
Male	10	83.33%
Ethnicity		
Asian	1	8.33%
White	11	91.67%
Years in policing		
1–2 years	6	50.00%
3–5 years	4	33.33%
6–10 years	1	8.33%
11–20 years	1	8.33%
Number of assaults		
1	1	8.33%
2	2	16.67%
3	5	41.67%
4	2	16.67%
5 or more	2	16.67%

M, mean; SD, standard deviation.

### Interview schedule

The interview schedule centered around five topics based on the research aims: types of assault that the participant had experienced; the impact of the assaults on the participant’s personal wellbeing; the impact of these assaults on the participant’s work; any differences between the assaults that the participant had experienced; and support provided to the participant following the assaults (see Appendix A for the full interview schedule). We used a semi-structured interview schedule to enable flexibility to explore topics during interviews. The interview schedule included open-ended questions and prompts to try to develop a conversational style that would elicit rich descriptions (Goldsmith et al., 2018).

### Procedure

Ethical approval was obtained from United Kingdom Health Security Agency Research and Governance Group, approval number: R&D 472. Participants were recruited through the Operation Hampshire network with invitations to participate advertised within participating police forces. Individuals that wanted to participate were asked to complete the screening questionnaire, the link to which was included in the advert. After the study had been advertised for a month and sufficient responses to the screening questionnaire had been received (*n* = 41) the screening questionnaire was closed, and selected participants were invited for interview. Some participants were removed for not fitting the criteria of two or more assaults in the past year (*n* = 5). Eligible participants were emailed to arrange an interview date. Interviews were conducted remotely over the online platforms Microsoft Teams or Skype between May and August 2022 and participants completed an online consent form prior to participating in the interview. Interviews were conducted by two researchers with previous experience in conducting interviews. Interviews ranged between 31 and 98 min, depending on the number of assaults that the participant had experienced. Each interview was recorded either within Microsoft Teams or with a dictaphone and then transcribed verbatim by an external company with whom the authors have a confidentiality agreement with.

### Data analysis

Data was analysed using framework analysis (Ritchie and Spencer, 1994) as it is grounded in data, flexible, and is particularly useful in research which has implications for policy (Pope et al., 2000). Two researchers read and re-read the interview transcripts to familiarize themselves with the data. They then met to develop a thematic framework which was created based on the initial read through of the interviews and the research aims. The thematic framework included: experience of assaults, impact on personal wellbeing, impact on work, support, factors exacerbating the impact of assaults, and impact of assaults on future assaults (see Table 2). Two researchers independently coded half of the data (six interviews each) using the qualitative analysis software, NVIVO 1.6.1, before meeting again to discuss the analysis. During this discussion, it was decided that factors exacerbating the impact of assaults, and impact of assaults on future assaults would be removed from the results section and instead focused on in the discussion because of the large overlap between these two themes and the other themes.

**Table 2:** Thematic framework for analysis.

Code	Description
1. Experience of assaults	
2. Impact on personal wellbeing	
Stress, anxiety, and negative emotions	The impact of the assaults on stress, anxiety, and negative emotions outside of work.
2.1 Sleep	Impact of assaults on participants ability to sleep.
2.2 Blame	Any blame placed on the participants for the assaults happening, either by themselves or by someone else.
2.3 Personal relationships	Any impacts of the assaults on participants’ relationships at home, e.g., with family, partner, friends etc.
2.4 Lasting physical pain	Physical pain that endures and impacts on participants’ day-to-day life.
2.5 Uncertainty	Uncertainty about what effect the assault will have on their health and future.
3.Impact on work
3.1. Confidence	Impact of assaults on participants’ confidence in approaching new situations and responding to calls.
3.2. Hypervigilance	Participants reported increased hypervigilance (i.e., scanning everything, heart rate going up, assessing possible scenarios, and trying to find solutions, being more wary).
3.3. Changed feelings towards the job	Participants reported negative feelings associated with working following the assaults (i.e., feeling more stressed and overwhelmed, worry about getting back to work, overthinking about the assaults, being anxious).
3.4. Working style	The changes to working style as an impact of the assault such as desk duty and time constraints.
4. Support
4.1. Continuing shift + time off	Participants reported they would have liked to take the shift off if given the option.
4.2. Lack of support	Participants reported lack of support following the assaults from both management and colleagues and not being aware/informed of services available.
4.3. Transparency of support	Participants not knowing what support is available to them.
4.4. Worry about reaching out	Participants reported worry about reaching out to their managers or asking for help with dealing with assaults mentally or for referrals, worrying if this could impact their jobs and what other would think about them.
4.5. Questioning if support would help	Participants unsure if support that is offered to them would help them.
5. Factors exacerbating the impact of assaults
5.1 Minority groups	Any unique impact on minority groups, such as race or gender.
5.2 Minimization	Participants reported assaults getting minimized, existing culture of minimization, joking about the assaults being the norm, penalization being insufficient etc.
5.3 Court proceeding	The impact of seeing their assaulter or waiting on investigations from the assault.
6. Impact of assaults on future assaults	

## Results

Four main themes and 12 sub-themes were identified during analysis: (i) experience of assaults; (ii) impact on personal wellbeing (stress, anxiety, and negative emotions; personal relationships; and physical effects); (iii) impact on work (confidence; hypervigilance; changed feelings towards work; and changed working style), and (iv) support (continuing shift and time off; support received; transparency of support; minimization of assaults; and worrying about reaching out).

### Experience of assaults

Participants were asked to discuss what happened during the assaults, which based on what participants responded can broadly be separated into two categories—physical and verbal. Physical assaults that participants experienced included being punched, kicked, slapped, scratched, headbutted, dragged/hit by car, and having items thrown at them. Physical assaults also included being bitten and spat at. The injuries that resulted from the physical assaults ranged from minor injuries (e.g., bruising and grazing) to more serious injuries (e.g., fractured bones and brain damage), as well as being contaminated with bodily fluids (e.g., saliva and blood). Verbal assaults included homophobic and racially aggravated insults being said to participants, often over a prolonged of time, for example one participant described the verbal assault against them lasting for up to an hour. Whilst participants explained the verbal abuse did not result in any physical injury, they did have a psychological impact on the participants.

### Impact on personal wellbeing

Participants were asked to discuss any ways in which the assaults affected their personal wellbeing, specifically, their physical and mental wellbeing. Within this theme, three sub-themes were identified: (i) stress, anxiety, and negative emotions, (ii) personal relationships, (iii) and physical effects.

#### Stress, anxiety, and negative emotions

Several participants discussed experiencing anxiety following the assaults, for example: “mentally, my anxiety, I do have days where I do not even want to get out of bed” (P1). Participants also described feelings of confusion and questioned why they had been assaulted, for example: “I remember just going to my partner constantly crying all the time thinking why me, why has it happened to me?” (P1).

Not being able to take their mind off the assault was a common concern that some participants had, for example: “unfortunately I have to think about these things on my days off when I should be relaxing” (P2).

In addition to having stress, anxiety, and negative emotions in the present, some participants also discussed how experiencing the assaults made them fearful because of the uncertainty of what might happen in the future, both in terms of being assaulted again, for example: “it absolutely petrifies me anything like that’s ever gonna happen again” (P4); but also, in terms of fearing for their health. This fear for their health was particularly prevalent among participants who had been spat at, or come into contact with another bodily fluid, for example: “I was just worried like, oh my God, what’s going to happen, has he got any diseases, am I going to get now any diseases, is that going to affect my personal life with my partner, everything just whizzes round in your head, so yes, that really affected me” (P1). Participants also described feelings of “disgust” that was specifically associated with spitting assaults.

Finally, several participants said that they had to attend court following the assault due to the perpetrator being charged for the assault. Participants spoke about how this impacted their mental wellbeing, for example some participants referred to the court process as a “stress” that was hanging over them (e.g., P2). Some participants distinguished between physical and verbal assaults stating because verbal assaults are more subjective the perpetrators tend to get a lesser sentence than a physical assault. One participant described this difference in length of sentence between physical and verbal assaults as “draining” because of the length it takes for a case to go to court (P3). A key part of the court process that participants spoke about that had a large effect on them was having to ‘re-live’ the assault and be questioned about it, for example: It feels horrible really, […] I’m not looking forward to it at all […] I’m not really fussed about seeing him again, it’s just re-living the incident as a whole and having someone say you are lying about something that you have been a victim of, it’s not nice” (P6).

#### Personal relationships

Several participants discussed the impact of the assaults on their personal relationships with family and friends outside of work. First, participants discussed not seeing friends outside of work after being assaulted due to them feeling like their friends did not understanding the impact that the assaults had on them, for example: “sometimes I isolate myself from friends who aren’t in the job because they will not understand what I’m going through” (P2). Participants also discussed struggling to communicate with friends due to anxiety created by the assaults, for example: “it also affected […] relationships […] outside of work because when I went into those anxious modes, I was really bad at communicating with family, friends, and everything” (P6).

When discussing the spitting assaults, one impact on participants personal relationships that was raised was the concern of passing any potentially contracted diseases onto family and friends. Because of this concern, some participants discussed not wanting to hug or kiss their family, for example: “they are long, long weeks where you are like not being able to kiss your partner or anything like that […] you cannot be affectionate towards people” (P4).

Finally, as well as negatively impacting their own wellbeing, some participants discussed the impact of the assaults on their partners wellbeing through causing their partners to worry about their safety personal safety when they are at work, for example: “and it impacted my family, my wife has feared for my safety everyday coming into work, and even though she knows that I’m in the process of […] becoming a police officer, that just makes her worry more” (P10).

#### Physical effects

Participants discussed the impact of the assaults on their physical wellbeing, such as experiencing physical pain following being assaulted. Just under half of the participants discussed experiencing long-lasting physical pain after being physically assaulted, for example: “I was still in a lot of pain, I still get pain now and then, so it’s always a constant reminder of what’s happened” (P1). Another participant said they experienced headaches for 3 months after being assaulted (P9). In terms of whether the physical or psychological effect of being assaulted had a greater impact on them, one participant said the physical impact had a greater impact on them because of the knock-on effects to other aspects of their life which was caused by the physical damage caused by the assault, for example: “it would have been the physical aspect, even though when I was back on desk duty, I had quite a big bandage on my hand for a while, and then my hand was weak or it was very tender so even doing simple things like picking up a bag with my right hand […] I would feel it sort of twinge, so yes […] I had to rethink everything for a bit” (P12).

### Impact on work

Participants were asked to discuss any ways in which the assaults impacted them at work, for example whether they took any time off after the assaults, whether the assaults affected their performance or confidence at work, and whether the assaults changed the way they felt about work. Within this theme, four sub-themes were identified: confidence, hypervigilance, changed feelings towards work, and changed working style.

#### Confidence

All participants discussed the impact that being assaulted had on their confidence at work. Some participants said that that the assaults affected their confidence in their own ability and made them question whether they were good enough for the job, for example: “making me not have confidence in myself, I already lacked confidence, but probably more so because I think am I doing this right?” (P1). Some participants expanded on this and said that their reduced confidence in their own abilities following the assaults impacted their work because it made them avoid certain situations, for example: “I was avoiding conflict at all costs because I wasn’t confident in my ability to handle it” (P6).

However, other participants said that the assaults did not impact their confidence in their physical ability to handle situations, but instead the assaults have reduced their confidence in their decision making, for example: “it definitely affected my confidence […] it did not affect my confidence in my own ability as such […] it’s my confidence in me making the decisions that aren’t then influenced by my fears of being assaulted and having someone be aggressive to me” (P11).

#### Hypervigilance

Several participants discussed experiencing feelings of hypervigilance at work following the assaults. Some participants described a general feeling of increased awareness of their surroundings, both when they are on shift, and also when they are not on shift, for example: “I now have the mindset of whenever I walk into any room, whether I’m on duty or off duty, I’m always looking for hazards, I’m always looking for, like the way out, where’s the door, […] it programs you into a robot this job” (P4). Other responders described specific ways that their behavior has changed due to this hypervigilance, for example, one participant said they change where they stand when they are in a room with other people: “it made me more kind of wary of people getting too close […] I now will not let people be out of my site, I will, depending on the situation I’m in, I’ll either have my back to the wall or so I can see who’s around me” (P10); and another participant said since the assault they are they are more ready to use their equipment: “it makes me stand back a bit more or realize more of my surroundings […] I’m extra cautious now with what happens, maybe hold my equipment more ready” (P1).

#### Changed feelings towards work

Participants spoke about how being assaulted changed the way they felt about being in the police. Some participants spoke generally about how some days they do not want to go in to work, for example: “I do have good days and I do have I cannot do this anymore, I do not really want to go to work” (P1). Other participants said since being assaulted, they have questioned whether they want to progress with their career in the police, for example: “I am looking to progress my job […] to become a police officer, at this current stage of my career, it did make me think” (P10). One participant said that they were unsure whether to work extra hours due to a fear of being assaulted again, for example: “I’m umming and ahing at the moment about doing overtime over summer […] what if I get assaulted again? I’m not scared of being assaulted; I do not want to be a victim again” (P3).

#### Changed working style

A key impact on participants’ work that they spoke about was how the assaults changed their working style. Participants discussed how the assaults psychologically changed the way they work, for example one participant spoke about becoming “overwhelmed” and “flustered” (P1) more easily which has impacted the way they work because they have to seek support and speak to their supervisors more regularly. Another way that the assaults changed participants working style was through making some participants quicker to judge or profile certain people into categories which subsequently has an impact on the way they treat them, for example: “I just automatically profile people in terms of […] you are being a nightmare, you are drunk, you are going to do it” (P4).

One participant said they have become less confrontational since being assaulted and this has changed the way they work because they try and prioritize calls that are not likely to be confrontational, and when they are with people who are aggressive, they are less likely to arrest them, for example: “If there was […] a call that we had to answer, that was likely to be less confrontational, I’d always prioritize going to that one, and when actually at jobs where it was confrontational […] I’ll try and play them down a little bit more when in reality I probably should have been sort of arresting people or being a bit more stern with people” (P6). Another participant said they are less likely to arrest people for public order offenses so they do not have to fill in paperwork and deal with the court proceedings, for example: “I just want to stop, because then I will not be a victim anymore, that’s why I do not like nicking anymore, even if someone is a dick in custody now, or shouting and screaming, I’ll just shout and scream back, no I do not nick, because I cannot be arsed with this” (P3).

### Support

Participants were asked to discuss any support that they received following the assault, for example from their employer or any external support. They were also asked to discuss whether they found this support helpful and whether they would have liked more support. Within this theme, five sub-themes were identified: continuing shift and time off, support received, transparency of support, minimization of assault, and worrying about reaching out.

#### Continuing shift and time off

All participants discussed whether they took time off following an assault or whether they continued at work. It was mixed across participants and assaults whether they took time off after the assault and this seemed to depend on the extent of their injuries and the type of assault. In particular, participants said they did not take time off after being spat at and said this was due to the perceived lack of severity of this type of assault. However, participants did not agree with this perception that being spat at is not a severe assault, for example: “if you have been assaulted, like been spat at and you and you have been told you could contract a disease […] what do you do? Well. you just […] sit at work […] and the emotions get worse because you are thinking about it” (P4).

Other participants said when they did take time off following an assault, they felt pressure to come back to work, even if they did not feel ready, for example: “I had 5 days off, it feels that I was pressured into coming back sooner, I would have liked to have a bit more time, but yes, then it did definitely feel like there was pressure to me coming back sooner rather than later because of general numbers […] I know that I could have turned round and said no, but then when you are getting the repeat phone call it just feels like your first no wasn’t good enough and then you have to justify your second and your third and so on, and then I gave in really” (P12).

However, other responders said they did not feel like they needed to take time off following an assault, for example: “I think to a degree […] it’s like falling off of a bike, if you do not get back on the bike you’ll probably always have that fear of getting back on the bike if that makes sense, I think if I’d had any time off it probably would have created more fear of going back to that kind of working style” (P10).

#### Support received

It was mixed between participants whether they received any support following their assaults. Some participants said they did receive support, and this was enough for them to be able to move forwards from the assault. Participants said this support came from either their line managers, for example: “my sergeants were supportive […] they made it […] as easy as they could for me” (P12), or from their peers, for example: “luckily I had a good colleague on my shift who has got about 20 years in the job so luckily she […] managed to kind of calm me down” (P4).

Some participants spoke about Trauma Risk Management (TRiM)—a trauma-focused support system designed to help people who have experienced a traumatic event. Some participants were offered TRiM but chose not to take it. Yet, other participants who were offered TRiM said it helped to support them following the assaults. One participant discussed how not only did TRiM directly help them following the assaults, but it also helped their line manager provide support, for example: “[TRiM] were highlighting the areas which it clearly affected me, and then that was fed back to my sergeants who were then able to make sure that I do not go to anything that’s going trigger that” (P12).

However, several participants said that they did not receive any support following an assault when they felt they needed it and would have liked it, for example: “I literally got nothing, no-one even contacted me to say that they are dealing with the case […] I had no one check in on me to make sure I was okay after the assault” (P1). Several participants said they felt let down due to the lack of support they received. One participant said they still did not receive any support even when they reached out and asked for it, for example: “and the counseling system, I tried speaking to them […] and they were like yes, we’ll get you on the space. I was like okay, and then they never called me back, so right now it’s just again, there’s no point in contacting them because they will not phone me back” (P3).

#### Transparency of support

Several participants said that following the assault, they were not aware of what support was available to them, and because of this, some participants said they were reluctant to reach out and ask for support, for example: “maybe it’s like a phone call from them and instead of me phoning them because […] I would not know what they could do for me” (P2).

In terms of who they would expect to provide them with support, most participants said they would expect their line manager to provide them with support in the first instance, or to point them in the right direction of what support is available. However, despite this expectation, several participants said that their line manager either did not provide them with support, or did not provide them with information about what support was available to them, for example: “I wasn’t aware of what support was available and my skipper was a bit of a closed book and did not really offer me as much in terms of advice and support, so I was pretty much on my own” (P4). In the absence of guidance from their line managers, this participant said they reached out to colleagues for guidance and support instead, for example: “it’s something that I only found out because she told me it was available […] if I never spoke to her, I never would have knew that that support was […] available to me, which in my eyes is […] mind boggling” (P4).

However, some participants said their line managers were not withholding information about support from them intentionally, instead, they said it was because their line managers also were not aware of what support wase available, for example, “I think it might have been because they were not aware of it either, yes I spoke to both of them […] and neither of them have ever been assaulted to the degree that it affected them long-term” (P12). In addition, this participant recommended the development of an information pack with details of what support is available which can be provided to people after they get assaulted: “if there was some sort of information pack with everything to do after you have been assaulted, that would have been helpful.”

#### Minimization of assaults

Whilst some participants said their peers supported them following the assaults, other participants said their peers often minimized the seriousness of the assaults, for example: “it turns into a joke […] you have got Hep C now […] that might be funny to them […] he was trying to […] make a joke of it […], it reminded me of everything” (P4). Participants said verbal assaults were often minimized by their peers more than physical assaults, for example: “it’s easier for it to feel like a proper job if you have been physically assaulted […] if you walked into the office […] and are like oh my God I got punched in the face, people are like oh my God what happened? […] you’d get the comments that’d go did you duck […] but it tends to be people accept that and go, that’s a terrible thing, whereas with the public order and like the verbal abuse […] you’ll still get the … and?” (P3). Some participants said that amongst some of their peers being assaulted is thought of as part of the job, for example: “I do not know whether it’s the old we need to man up type of mentality that […] the older officers and the sergeants kind of see” (P4). In addition, some participants also said that the court system can also minimize the assaults, for example “the courts do not seem to take [being assaulted] seriously it’s almost like we are expected to take it, and I do not think anybody should be expected to be assaulted” (P8).

#### Worrying about reaching out

Some participants said that whilst they would have liked support, they did not feel like they were able to ask for it. Participants discussed different reasons for why this was. First, some participants said they felt unable to speak to their line manager about the assaults and ask them for support, for example: “I found it very hard to speak to my line manager about it because it, I felt like if I went to him and said I’m really struggling here, […] you need to deal with it because we pay you to work and I feel like that’s what he would have […] said” (P4). Second, one participant discussed how they were worried about the knock-on impact if it went on their record that they had received mental health support following the assault, for example: “what I’m worried about is that where I’ve previously suffered with my mental health before the job, I’m just worried that if I go and speak to welfare or mental health services or anything like that, it’ll go against me as a person and my track record” (P1). Finally, one participant said that because of an ongoing investigation into the assault, they felt unable to request support as they did not want to be seen to be influencing the investigation in anyway, for example: “but during the process I did not want to be seen to try and change the view of the people investigating me by going, oh I’m injured. I did not want to change their perception of the incident, they saw what had happened to me, they should take inference from that, so I remained quiet and being a relatively new officer, being under investigation was a terrifying time” (P9).

## Discussion

The current study aimed to investigate the impact of repeat assaults on police officers’ wellbeing and occupational outcomes. For this purpose, semi-structured interviews were conducted with police officers who had experienced repeat assaults by members of the public whilst at work in the last year. This study aimed to identify the impact of repeat assaults on police officers’ mental and physical wellbeing, as well as to identify the impact of repeat assaults on aspects of police officers’ occupation (such as job satisfaction and performance). In addition, this study aimed to explore how prior experiences of assault might influence subsequent experiences and explore factors which may exacerbate or aggravate the impact of repeat assaults. The discussion is structured around these research aims.

### Impact of repeat assaults on police officers’ mental and physical wellbeing

The impact of repeat assaults on police officers’ mental and physical wellbeing was a key focus for participants throughout the interviews. Many participants reported lasting physical pain after being assaulted, with some participants reporting continued health concerns following assaults where they were contaminated with bodily fluids. For example, a significant number of police officers reported having to have their bloods taken for months following the assault due to ongoing infection concerns. In addition, several participants reported an increase in negative emotions during the period following the assaults (e.g., anxiety, depression, stress, fear, uncertainty), as well as high levels of overthinking and replaying the experience. Importantly, reported negative feelings were not only a result of the assault itself, but also of different aspects surrounding the assaults, such as having to go to court and deal with court proceedings. Participants’ personal relationships with family and friends outside of work were also affected by the assaults. For example, some participants reported feeling withdrawn and avoiding contact with friends and family following the assaults, while in cases of possible contamination the behavior changes were even more significant, with many officers reporting avoiding physical contact with their partners and family members.

Findings are in line with previous research which report the negative impact assaults have on police officers’ wellbeing. For example, research has shown that violence and injuries sustained in the line of duty led to increased perceived stress, feeling worn out and tired, emotional exhaustion, and increased psychological distress among police officers (Santos et al., 2009; Leino, 2013; West et al., 2017; Wolter et al., 2019). Furthermore, Karlsson and Christianson (2003) found that police officers exposed to traumatic situations often experienced long-lasting depression, fear, guilt, tension, feelings of withdrawal, irritability, and nightmares. Finally, legal proceedings surrounding assaults have been found to put police officers at increased risk for posttraumatic stress disorder (Ellrich and Baier, 2017). This finding that legal proceedings can exacerbate the impact of assaults on police officers’ mental wellbeing is in line with the current study which found that participants’ reported stress, anxiety, and generally negative emotions in their accounts of dealing with court proceedings following being assaulted.

### Impact of repeat assaults on police officers’ occupation

Participants discussed the impact being assaulted had a on their confidence. For example, following the assaults most participants said their confidence in their own abilities and their confidence in suitability for carrying out their job decreased. Participants also discussed the impact that being assaulted had on their feelings towards their job. For example, several participants reported feelings such as a lack of motivation and commitment to their work, disappointment in the way the assault had been handled by their managers or the courts, stress whilst at work, and job dissatisfaction. Furthermore, participants also reporting conflicting emotions regarding the perceived constant risk to their health and wellbeing they face whilst at work compared to the perceived benefits. Some participants even indicated a desire to leave their force after being assaulted. The findings discussed in the current study regarding the impact the repeat assaults had on participants occupation as police officers are in line with findings from previous research which found that workplace assaults can reduce job investment (Van Emmerik et al., 2007) whilst also increasing job stress, which in turn can reduce job satisfaction and commitment (Manzoni and Eisner, 2006).

Also in relation to the impact of repeat assaults on participants job was the hypervigilance to surroundings that several participants reported, as well as behavioral changes in their working style. For example, participants reported an increase in hypervigilance-related cognitive tendencies, such as experiencing increased feelings of suspicion, mistrust, and negative expectations for the future after being assaulted, compared to before. In addition, some participants also reported behavioral and physiological symptoms associated with this increased hypervigilance following assaults, such as an increase in safety-keeping behaviors (e.g., planning escape routes, making sure there is adequate coverage and protection, and avoiding situations requiring conflict), as well as an increase in physiological arousal (e.g., increased heart rate). This finding is in line with previous research that found that hypervigilance appears to be a cognitive, physiological, and behavioral pattern (Ehlers and Clark, 2000; Ehring et al., 2008; Kimble et al., 2013) and can predict posttraumatic functional impairment (Norman et al., 2007). Thus, a possible explanation for the increase in hypervigilance-associated symptoms experienced by participants in the current study could be due to the *anticipation* of future assaults. Accordingly, Leino (2013) found that the frequency of work-related violence among police officers is associated with greater psychological distress and fear of future assaults. With this in mind, the consequences of hypervigilance are important to consider when designing and tailoring effective support systems for police officers following repeat assaults.

### Impact of past assaults on future assaults

Given the impact identified in the current study of repeat assaults on participants behavior and working style, it follows logically that the current study found that the experience of being assaulted also affected the way that participants dealt with future assaults. For example, as discussed above, in accordance with the presence of hypervigilance symptoms, many participants had marked changes in their working styles, which in turn affected the way the approached future situations. For example, after being assaulted, many participants reported a change in working style towards more protective type behaviors in order to either try and prevent any potential confrontations between themselves and an offender, or if a confrontation has taken place, in order to try and minimize any potential harm to themselves that might occur as a result of the confrontation. Examples of these protective type behaviors discussed by participants included being less patient with offender, being more aware of their surroundings, making sure they were protected and visible to witnesses, and expecting certain behaviors from offenders. However, not all participants reported the negative impact of assaults on future behavior, some discussed learning from being assaulted and how to change their behavior in a positive way going forwards into similar situations.

### Factors exacerbating the impact of repeat assaults

The final key research aim was to identify any factors that potentially exacerbated the impact of repeat assaults, accordingly support—or lack of—that participants received following the assaults has been identified as a key factor. Several participants reported receiving little or no support after being assaulted, despite wanting and feeling like they needed it. Furthermore, several participants also reported being unaware of what support was available to them. In addition to the lack of support participants discussed receiving, several also reported feeling frustrated and disappointed about having to continue at work after being assaulted and were not given an option to take time any off work. These findings are in line with previous research that found that police officers generally perceived support services available to them within their department as inadequate (McMurray, 1990). However, the findings from the current study go even further than this by suggesting that not only the support systems available to participants were perceived as inadequate, but also at times were not available. This finding is particularly important as research suggests that receiving support evidence-based treatment following a traumatic exposure is an important mitigator for post-traumatic stress disorder (McFarlane and Bryant, 2007). Furthermore, research suggests that the employment of the internal resources to support employees is more effective in preventing psychiatric work disability than the utilization of the external health care system (Weisæth and Kjesrud, 2007), thus placing even greater importance on the provision of support to police officers within the organization. Accordingly, the provision of preparatory sessions on assaults has also been offered as an effective way to mitigate the impact of assaults as they increase perceptions of control and competence (Ellrich and Baier, 2017).

In the current study, participants not only commented that they did not receive support from their line managers, but some participants also said they did not receive the amount of support they would expect from their colleagues following the assaults with participants reported that colleagues would often minimize the potential impact of assaults on their wellbeing and said some of their colleagues would not take them seriously. This is an interesting and important finding because research has shown that a lack of social support work can reduce police officers’ ability to cope with stress as well as their willingness to be involved with their job (Lord, 1996). Furthermore, recent research has identified peer support has been identified as a coping mechanism for posttraumatic stress disorder among police officers (Foley et al., 2022). Thus, based on these findings that support (both organizational and peer) can reduce potential negative impacts of assaults, as well as exacerbating them when support is not present emphasizes the importance of providing adequate support to police officers after they have been assaulted as well as making sure that any potential support that might be available to police officers is made known to them.

Yet, in addition to organizational and peer support reducing potential negative impacts of assaults on police officers’ well-being, recent research has shown that perceived social support from family and significant others can also reduce negative impacts on police officers’ well-being (Padhy et al., 2022). In the current study some participants reported feeling isolated from their family and significant others and not being able to be intimate with them. Thus, this lack of interaction and opportunity to receive support from people who are likely to be able to help mitigate any negative effects on participants wellbeing could exacerbate any potential negative impacts of the assaults.

### Strengths and limitations

To the best of our knowledge, this is the first study that investigates the impact of repeat assaults on police officers’ wellbeing. The in-depth nature of the interviews provides a detailed and unique perspective on the impact of repeat assaults on police officers’ wellbeing and highlights several important aspects of their lives that were affected, such as mental and physical health and occupation. Furthermore, the study also provides an important and current perspective on the support services and the procedures currently in place following assaults on police officers, whilst also identifying existing barriers and factors exacerbating the impact of the assaults, and important facilitators for tailoring more effective support systems.

However, despite the strengths of the research, there are limitations that need to be addressed. First, whilst efforts were made to diversify the sample by inviting a wide range of potential participants to take part, the final sample represented in just two participants being female. Even though the prevalence of female officers in the police force in the United Kingdom is not equal to the number of males, the current study still falls short of the national average of 35.5% female officers in England and Wales, according to Statista (2022). Furthermore, the majority of participants in the study had less than 5 years’ experience in service. It has recently been highlighted that younger police officers experience more fear when they are working, whereas older police officers are more fearful of being negatively appraised when at work (Sundaresan and Sharma, 2022). Therefore, based on this lack of diversity with both gender and years of experience among participants, the generalizability of the sample to the wider police population may be limited and it is difficult to discern whether the findings identified in the study are representative of the wider population, or whether they represent the particularities of the participants that took part. However, whilst the sample size does seem to lack diversity, consistent themes did arise across participants therefore we can be fairly confident that our findings are relevant to the wider police population. Yet, it would be useful for future research include a more diverse sample set in order to strengthen the confidence in which any conclusions can be made.

A second limitation is the seemingly low sample size of just 12 participants, given the relatively broad and exploratory nature of the research aims. There is often a debate in qualitative research around data saturation—defined by Guest et al. (2006) as “the point in data collection and analysis when new information produces little or no changes to the codebook” (p.65; see Braun and Clarke, 2021 for a review)—and at what point researchers should stop collecting data. Data collection in the current study ceased due to no new participants volunteering and potential participant pools for recruitment being exhausted, rather than data saturation and thus potential themes may have been missed. However, consistent themes were identified across several participants who varied in the range of assaults they had experienced. Therefore, whilst specific factors linked to more unique assaults may have been missed, we can be confident that the factors identified in the current study are representative of wider assault situations. Yet, to strengthen our confidence in the representativeness of identified factors, future research would benefit from further investigating the impact of repeat assaults on police officers.

## Conclusion

Taken together, the findings suggest experiencing repeat assaults can have a negative impact on many areas of police officers’ wellbeing, including physical and mental wellbeing, as well as impacting their relationships with friends and family, and their perceived ability and motivation to do their job. Findings highlight the current perceived lack of support that police officers are provided with after being assaulted as well as sometimes a lack of support from peers due to the impact of assaults being minimized by colleagues. When support is available to police officers this can act a s a mitigating factor to the negative impacts of assaults on police officers’ wellbeing. Therefore, we emphasize the importance of the provision of support following an assault, as well as transparency in what support is available so police officers can seek further support if they require it. Given the finding that being assaulted can change police officers’ behavior and make them more hostile, protective, or hesitant during future potential violent encounters, the provision of early support is essential to mitigate any potential impacts of prior assaults on future assaults. Taken together, the findings from the current study provide a unique and in-depth perspective behind police officers’ experiences following repeat assaults, which can in turn inform national policies and help tailor effective support services within the police force.

## Data availability statement

The data that support the findings of this study are available on reasonable request from the corresponding author. The data are not publicly available due to ethical restrictions.

## Ethics statement

Ethical approval was obtained from United Kingdom Health Security Agency Research and Governance Group, approval number: R&D 472. The patients/participants provided their written informed consent to participate in this study.

## Author contributions

AD and LD are the joint lead authors. All authors contributed to the article and approved the submitted version.

## Conflict of interest

The authors declare that the research was conducted in the absence of any commercial or financial relationships that could be construed as a potential conflict of interest.

## Publisher’s note

All claims expressed in this article are solely those of the authors and do not necessarily represent those of their affiliated organizations, or those of the publisher, the editors and the reviewers. Any product that may be evaluated in this article, or claim that may be made by its manufacturer, is not guaranteed or endorsed by the publisher.
