# Supplementary material for: “I don’t want to be a victim again”: the impact of repeat assault on police officers
Source: Front Psychol. 2023 May 18;14:1145944. doi: 10.3389/fpsyg.2023.1145944 (PMC10232816; doi:10.3389/fpsyg.2023.1145944)
Supplement: Supplementary file 1 [file Data_Sheet_1.docx]

**Appendix A.** Interview schedule

**Op Hampshire Interviews: Understanding the Impact of Repeat Assaults in Policing**

**Interview Guide**

***Introduction***

[*Before starting the interview, the following script should be approximated. Additionally, the location of the interviewee should be recorded in the case that the Clinical Risk and Safeguarding Protocol is required. This location should be handwritten on a blank piece of paper and destroyed once the interview is completed.]*

Thank you for joining me for an interview today.

I am a researcher from the UK Health Security Agency, working on a project in collaboration with the National Police Wellbeing Service and King’s College London, aiming to understand the impacts of repeat assaults in policing. As part of this research I am conducting interviews with police officers and staff who have experienced repeated assaults by members of the public in the workplace in the past year. During the interview I will ask you questions about the assaults that you experienced, the impact on your wellbeing, the impact on your work, and about any support you were provided following the incidents. The interview will last up to 2 hours.

Your participation in this research is completely voluntary, and you are under no obligation to take part. You are free to withdraw from the study or to stop or pause the interview at any time.

All of the information you provide will be anonymous and confidential to us. We will not identify you to anyone else in the course of our research. To maintain anonymity, we ask you not to state your name or any other information that could be used to identify you during the interview. But, if you do accidentally say anything that could be used to identify you, this will be removed during transcription.

The only exception to confidentiality is if you disclose any information to me indicating risk to your safety or the safety of others, or about a previously non-disclosed serious crime that you or someone else has committed or is about to commit, in which case I will be legally required to disclose this information to the relevant authorities. If this happens, I will discuss this with you to ensure that you understand what I am going to do beforehand.

I will be audio recording the interview, and will get your permission before I start the recording.

Before I start the recording, I would also like to ask you for your current location. This is so we have a record during the interview for safeguarding, it will not be recorded as part of the interview and will be securely destroyed at the end of the interview.

Before we start do you have any questions you want to ask me?

We’re almost ready to start the interview. Are you happy for me to start recording?

*[Start audio recording]*

***Verbal Consent***

- Are you happy for me to interview you about your experiences of repeat assaults in the workplace?
- Do you understand that you can ask me to stop or pause the interview at any time?
- Do you understand that the information you provide will be anonymous and confidential, the only exception being if you disclose any information that I would be legally required to disclose to the relevant authorities?

***About the Assaults***

1. Can you tell me a little bit about your job role?
2. In the questionnaire you completed earlier, you said you had experienced [X] assaults in the workplace in the past year. Can you tell me a bit about each of these assaults?

Prompts:

- What happened?
- When did the assault(s) happen?

***Impact on Work***

1. Can you tell me any ways in which these assaults impacted you at work?

Prompts:

- Did you take any time off work after any of the assaults?
- Have they affected your performance at work?
- Have they affected your confidence at work?
- Have they changed the way you think or feel about your work?
- What aspects of the assaults have most affected your work?

***Impact on Wellbeing***

1. Can you tell me any ways in which these assaults affected your personal wellbeing?

Prompts:

- If so, How have they affected your physical wellbeing?
- If so, How have they affected your mental wellbeing?
- If so, What aspects of the assaults have most affected your mental wellbeing?

***Differences Between Incidents***

1. Are there any important differences between the ways that each of the assaults have affected you?
2. Do you think your prior experiences of assaults shaped your subsequent experiences in any way?

***Support Provided***

1. Can you tell me about any support that you received following the incidents?

Prompts:

- Did you receive any support from your line manager?
- Did you receive any support from your employer?
- Did you receive any external support?
- Did you find the support helpful?
- Would you have liked to receive more support?

***Other***

1. Is there anything else that you would like to comment on that we haven’t already discussed?
